# Pathways Exploited by Flaviviruses to Counteract the Blood-Brain Barrier and Invade the Central Nervous System

**DOI:** 10.3389/fmicb.2019.00525

**Published:** 2019-03-28

**Authors:** Yasmin Mucunã Mustafá, Lana Monteiro Meuren, Sharton Vinícius Antunes Coelho, Luciana Barros de Arruda

**Affiliations:** Departamento de Virologia, Instituto de Microbiologia Paulo de Góes, Universidade Federal do Rio de Janeiro (UFRJ), Rio de Janeiro, Brazil

**Keywords:** blood-brain barrier, flavivirus, Japanese encephalitis virus, West Nile virus, Zika virus, dengue virus, yellow fever virus, brain microvascular endothelial cells

## Abstract

Human infection by different flaviviruses may cause severe neurologic syndromes, through pathogenic mechanisms that are still largely unknown. Japanese encephalitis virus (JEV), West Nile virus (WNV), Zika virus (ZIKV), yellow fever virus (YFV), dengue virus (DENV), and tick-borne encephalitis virus (TBEV) are believed to reach the central nervous system by a hematogenous route, upon crossing the blood-brain barrier. Although the disruption of BBB during flavivirus infection has been largely evidenced in experimental models, the relevance of BBB breakdown for virus entering the brain was not completely elucidated. *In vitro* models of BBB had demonstrated that these viruses replicated in brain microvascular endothelial cells (BMECs), which induced downregulation of tight junction proteins and increased the permeability of the barrier. Other reports demonstrated that infection of BMECs allowed the basolateral release of infectious particles, without a remarkable cytopathic effect, what might be sufficient for virus invasion. Virus replication and activation of other cells associated to the BBB, mostly astrocytes and microglia, were also reported to affect the endothelial barrier permeability. This event might occur simultaneously or after BMECs infection, being a secondary effect leading to BBB disruption. Importantly, activation of BMECs, astrocytes, and microglia by flaviviruses was associated to the expression and secretion of inflammatory mediators, which are believed to recruit leukocytes to the CNS. The leukocyte infiltrate could further mediate viral invasion through a Trojan horse mechanism and might contribute to BBB breakdown and to neurological alterations. This review discussed the previous studies regarding *in vitro* and *in vivo* models of JEV, WNV, ZIKV, YFV, DENV, and TBEV infection and addressed the pathways for BBB overcome and invasion of the CNS described for each virus infection, aiming to increment the knowledge and stimulate further discussion about the role of BBB in the neuropathogenesis of flavivirus infection.

## Introduction

Flaviviruses encompass arthropod transmitted viruses, which may cause systemic, hemorrhagic, and neurological syndromes. Japanese encephalitis virus (JEV), West Nile virus (WNV), Zika virus (ZIKV), yellow fever virus (YFV), dengue virus (DENV), and tick-borne encephalitis virus (TBEV) are some of the most prevalent flaviviruses associated to human diseases in different regions of the world. Most of the individuals affected by those infections develop asymptomatic or mild manifestations, but these viruses may cause severe neurologic syndromes, once they reach the central nervous system (CNS).

The frequency of neurological manifestations upon flavivirus infection varies considerably depending on the virus. Severe infection by JEV, WNV, TBEV, and congenital ZIKV is associated to encephalitis and other neurological syndromes (Lindquist and Vapalahti, 2008; Melo et al., 2016; Salimi et al., 2016; Samy et al., 2018), whereas the severe disease caused by DENV and YFV are more related to vascular and systemic manifestations (Jennings et al., 1994; Monath and Barrett, 2003; Beck and Barrett, 2015; Halstead and Cohen, 2015; Screaton et al., 2015; Li et al., 2017). Even though these viruses usually do not affect the CNS, a recent surveillance study performed in Brazil reported the presence of laboratory markers of DENV and ZIKV in patients with acute neurological diseases. Among 74 studied cases, 2 patients presented DENV RNA in the CSF and 6 patients showed anti-DENV IgM in the serum. Also, three patients showed ZIKV RNA or IgM in the CSF or in the serum. These data suggest that arboviruses may play a more important role in CNS-associated disease than previously found, at least in endemic countries where the number of infected individuals is very high (Vieira et al., 2018).

All the referred viruses are able to infect neurons, albeit they are not always neuroinvasive (Jennings et al., 1994; Ramos et al., 1998; Chen et al., 2012; Hussmann et al., 2013; Garcez et al., 2016). Viruses can reach the brain through different mechanisms, including peripheral nervous system and axonal transport, and hematogenous route. Although experimental models had previously indicated that some flaviviruses, such as WNV and JEV, disseminated *via* axonal transport or through the olfactory bulb, those models were developed using intrasciatic and intranasal inoculation (Samuel et al., 2007; Yamada et al., 2009), which may not represent their natural infection route. Given the systemic nature of flavivirus infection, and since a disruption of the blood-brain barrier (BBB) is often observed in experimental models of infection, it is believed that crossing this structure is a major mechanism for flavivirus neuroinvasion (Verma et al., 2010; Jurado et al., 2018; Wang et al., 2018).

**Figure 1:**
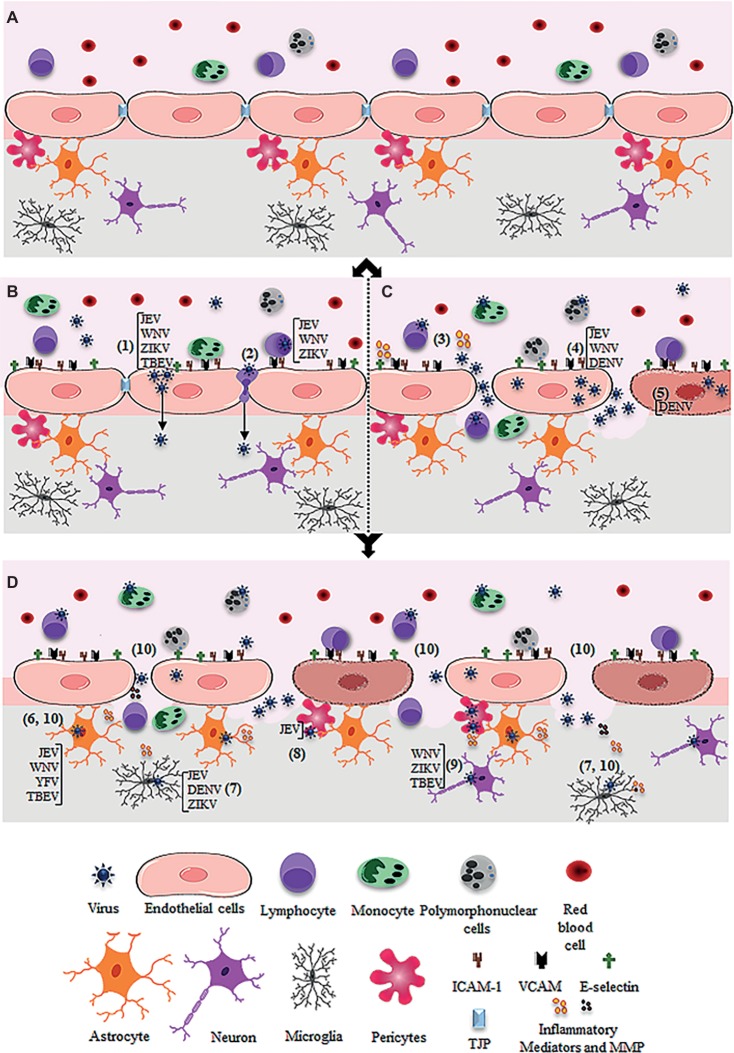
Schematic figure showing possible mechanism of virus entry into the central nervous system through the blood-brain barrier. **(A)** Intact blood-brain barrier is composed by endothelial cell strongly adhered through tight junction proteins (TJP), in association to pericytes, astrocytes, and microglia. The barrier controls the flux of solutes, blood cells (lymphocytes, monocytes, and polymorphonuclear cells), and pathogens from the blood to the central nervous system. **(B)** After systemic infection, some flavivirus reaches the BBB through hematogenous route and may cross the endothelial barrier without remarkable cytopathic effect. JEV, WNV, ZIKV, and TBEV may cross the endothelial barrier as cell-free virus **(1)**. JEV, WNV, and ZIKV were also reported to traverse the endothelial barrier associated to infected leukocytes **(2)**. **(C)** Systemic infection and inflammation and/or the direct infection of brain endothelial cells may induce BBB breakdown, allowing virus invasion of the CNS. Regarding this, systemic inflammation due to activation of immune cells upon infection was associated to the release of inflammatory mediators, which then affect the permeability of the endothelial barrier **(3)**. Also, replication of JEV, WNV, and DENV in the brain endothelial cells may induce downregulation of TJP expression **(4)** and/or cell death **(5)**, promoting the barrier disruption and virus entry. **(D)** After entry into the brain by any of the described pathways **(B** and **C)**, flaviviruses may infect the astrocytes (as described for JEV, WNV, YFV, TBEV) **(6)**, microglia (as described for JEV, DENV, ZIKV) **(7)**, pericytes (as described for JEV) **(8)**, and neurons (as described for WNV, ZIKV, TBEV) **(9)**. The infection of these cells, especially astrocytes and microglia, induces the release of inflammatory mediators (IL-6, VEGF, TNF-α, IFN-γ, IL-1β and IL-10, MCP-1) and metalloproteinases (MMP2, MMP3, MM9) **(10)**, which mediate the downregulation of adherents and tight junction proteins (TJP), resulting in increased permeability.

The BBB is a complex structure, composed by tightly adhered brain microvascular endothelial cells (BMECs), which are associated to pericytes, astrocytes, and microglia. This structure constitutes a barrier between the blood and the CNS parenchyma, acting in the flux regulation of solutes, cells, and pathogens (Figure 1A; Ballabh et al., 2004). The cells associated to the BBB express a collection of pattern recognition receptors (PRR) and are able to respond to PAMPs derived from pathogens and to DAMPs released upon CNS damage (Zamanian et al., 2012; da Conceição et al., 2013; Minkiewicz et al., 2013; Wan et al., 2014). Therefore, an inflammatory insult or other pathobiological conditions that affect their metabolism may impact the barrier function (Argaw et al., 2012; Sofroniew, 2015).

BMECs are unique unfenestrated polarized endothelial cells, connected by continuous tight junctions (TJs). The junctions are composed of TJ proteins – claudins and occludin, and of adhesion molecules – E- and VE-cadherins. The TJ complex is stabilized by its association to intracellular cytoskeleton through adaptor proteins, such as zonula occludens (ZO), among others (Chow and Gu, 2015). Those features, in association to the low rate vesicular trafficking of the brain endothelial cells, turn them more resistant to paracellular and transcellular trafficking, in comparison to endothelial cells from other tissues (Obermeier et al., 2013; Siegenthaler et al., 2013; Chow and Gu, 2015). In addition, BMECs express low levels of leukocyte adhesion molecules, limiting the infiltration of immune cells into the brain (Siegenthaler et al., 2013). Virus infection of BMECs may result in cell death or in decreased expression and organization of TJ proteins, directly impacting the integrity of the endothelial monolayer. The activation of the BMECs due to virus replication may also promote the production of mediators that affect the BBB structure and stimulate leukocyte recruitment, including cytokines, such as IL-6 and TNF-α, reactive oxygen and nitrogen species (ROS, NO), and prostaglandins (Schreibelt et al., 2007; da Conceição et al., 2013; Rochfort et al., 2014; Papa et al., 2017; Soe et al., 2017).

BBB endothelial cells are surrounded by the basement membrane, and the structure and function of this core are supported and regulated by adjacent pericytes, astrocytes, and microglia (Chow and Gu, 2015). Astrocytes endfeet covers most of the CNS vasculature, connecting it to the neuronal parenchyma. Therefore, mediators produced upon BMEC activation or after CNS damage affect the function of astrocytes. Also, these cells may be directly infected and activated by viruses (Hussmann et al., 2013; Chang et al., 2015). All the cited stimuli promote a response called astrogliosis, and reactive astrocytes produce cytokines, chemokines, prostaglandins, and nitric oxide (Argaw et al., 2012; Zamanian et al., 2012; Sofroniew, 2015). These mediators modulate angiogenesis, affect TJ proteins expression and organization, restrict or stimulate leukocyte infiltration, and impact neuronal function.

Hence, insults like virus infection and inflammation can stimulate BMECs, astrocytes, and other cells to secrete mediators that will ultimately affect the BBB integrity and CNS function (Fabry et al., 1995; Argaw et al., 2012; Heinemann et al., 2012; Alvarez et al., 2013).

Given the systemic nature of flavivirus infection and the physiological function of the BBB, it is generally believed that BBB disruption is a determinant event preceding viral invasion. A major point supporting this conception is that BMECs, which are the main cell types in the BBB and are susceptible to all neurotropic flaviviruses. Virus replication often induces apoptosis, and several studies suggested that neuroinvasion involved infection of BMECs (Wang et al., 2008a,b; Verma et al., 2009; da Conceição et al., 2013; Lazear et al., 2015; Al-Obaidi et al., 2017; Papa et al., 2017; Patabendige et al., 2018). It is important to notice, however, that due to technical issues inherent to analysis of brain tissues, there are very few studies confirming the infection of BMECs upon human natural infection (Johnson et al., 1985; Ramos et al., 1998). The great majority of the studies were done *in vitro*, with transformed cell lines (da Conceição et al., 2013; Patabendige et al., 2018). Also, since wild-type adult mice were resistant to infection, most of the studies performed *in vivo* used immunodeficient or suckling mice (Velandia-Romero et al., 2012; Daniels et al., 2017; Papa et al., 2017). Therefore, although flavivirus replication in BMEC has been largely demonstrated in experimental models, further analyses of human tissues are critical to further support this hypothesis.

Increasing evidence suggest that flaviviruses can also reach the CNS by crossing the BBB, without barrier breakdown. The basolateral release of infectious particles upon virus replication in BMECs was described in different experimental models, in which no significant cytopathic effect (CPE) had been detected (Verma et al., 2009; Papa et al., 2017). Virus crossing by transcytosis in brain endothelial cells had also been reported (Liou and Hsu, 1998; Papa et al., 2017) and may allow free virus to reach and infect the neurons. Flavivirus may also reach the CNS as cell-associated particles, through a mechanism called “Trojan horse” (Verma et al., 2009). In this context, peripheral-infected leukocytes transmigrate through the endothelial cell layer and release virus within the CNS. The released infectious particles can then infect resident cells. In healthy conditions, the BBB restricts leukocyte infiltration. Therefore, virus invasion through a Trojan horse mechanism requires either a rupture in the BBB structure or alterations of the TJ that allows paracellular migration of the cells. Activation of brain endothelial cells, with increased expression of adhesion receptors and enhanced secretion of chemokines, is also required to attract the infected leukocytes to the brain.

Viral invasion of CNS by all these mechanisms is often followed by leukocyte infiltration. Both virus replication in neurons and the local inflammatory response triggered afterward were reported to be relevant in the neuropathogenesis of flavivirus infection (Roe et al., 2012; Velandia-Romero et al., 2012; Li et al., 2015; Douam et al., 2017; Jurado et al., 2018; Lucas et al., 2018; Wang et al., 2018). Subsequent inflammation elicited after virus invasion of the CNS may also contribute to BBB breakdown and amplify the whole process.

In this review, we will address whether the flaviviruses such as JEV, WNV, ZIKV, YFV, DENV, and TBEV infect BMECs and whether this infection is relevant for the release of infectious particles, able to disseminate through the CNS, and for the disruption of BBB. We will also consider if the infection of other cell types associated to the BBB, such as pericytes, astrocytes, and microglia, triggers cellular activation and the role of this secondary effect for BBB disruption. Finally, we will discuss whether BBB disruption is indeed essential for virus dissemination into the brain.

## Japanese Encephalitis Virus

Japanese encephalitis virus (JEV) is an important virus agent causing neuropathological disorders in Asia, Australia, and Western Pacific (Samy et al., 2018). Although most individuals infected by JEV present subclinical or febrile mild infection, a small proportion of patients develop encephalitis or meningoencephalitis, which is often fatal or may lead to permanent motor or cognitive deficits (Salimi et al., 2016). Neuroinfection is characterized by disruption of the BBB and extensive inflammation in the brain (Bian et al., 2017; Wang et al., 2018). However, the pathway of JEV entry into the CNS and mechanisms associated to the inflammatory response are not completely elucidated.

JEV is able to infect a variety of cell types present in the BBB, including BMECs, microglia, astrocytes, and pericytes, and *in vitro* infection models have been exploring the effect of viral replication on BBB permeability (Chen et al., 2012,^2014^; Chang et al., 2015). Infection of BMECs by JEV did not affect cell viability but resulted in increased permeability of the endothelial monolayer (Lai et al., 2012; Al-Obaidi et al., 2017; Patabendige et al., 2018), suggesting that virus-induced cell death is not the mechanism responsible for BBB disruption and viral invasion. Accordingly, JEV infection of multiple cell types diminished the expression and altered the localization of adherents and tight junction proteins. This effect was also observed when the cells were cultured with virus capsids, instead of infectious particles, suggesting that altered endothelial permeability may happen even in the absence of productive viral replication (Agrawal et al., 2013).

Other studies, however, reported that JEV infection promoted negligible effect on transendothelial electrical resistance (TEER) of rat-derived isolated BMEC, unless they were cultured with other cell types associated to the BBB (Chen et al., 2014; Chang et al., 2015). Among those cells, pericytes and astrocytes are both permissive to JEV replication. Infection of these cells, *in vitro*, induced the release of soluble mediators that degrade tight junction proteins, particularly zonula occludens-1 (ZO-1) (Chen et al., 2014; Chang et al., 2015). Indeed, addition of pericytes or conditioned medium derived from infected pericytes to a culture of BMECs induced endothelial permeability, in a pathway partially dependent on secreted proteases and IL-6 (Chen et al., 2014). Similarly, culture of BMECs with conditioned medium obtained from JEV-infected astrocytes induced the degradation of ZO-1 and claudin-5 and BMEC permeability, in a way dependent on IL-6, VEGF, and metalloproteinases (MMP-2/MMP-9) (Chang et al., 2015).

An *in vitro* model of human BBB, using human BMECs (HBMECs) and astrocyte cell lines, also demonstrated that both cell types were susceptible to JEV (Patabendige et al., 2018). The interaction of endothelial cells with astrocytes was necessary for JEV-induced BBB permeability, and it was associated to increased secretion of inflammatory cytokines. Microglial cells were also activated upon JEV infection *in vitro* and produced inducible nitric oxide synthase (iNOS), IL-1β, IL-6, MCP-1, and TNF-α (Thounaojam et al., 2014), what may contribute to endothelial barrier dysfunction. According to these studies, JEV-induced BBB breakdown seems to be rather a bystander event than to direct virus replication in BMECs. However, JEV interaction with the endothelial cells could initiate the process, by allowing virus access to pericytes, astrocytes, and microglia, which would, then, secrete mediators that degrade the tight junctions and disrupt the barrier.

Mouse and primate *in vivo* experimental models supported that JEV infection promoted BBB breakdown, leukocyte infiltration, and activation of astrocytes and microglia (Myint et al., 2014; Li et al., 2015; Wang et al., 2018). BBB disruption due to decreased expression of ZO-1 and claudin-5 was detected in JEV-infected mice at the time point when neuropathological syndrome was evident (Li et al., 2015; Wang et al., 2018). In addition, the presence of activated astrocytes and microglia and a robust production of inflammatory cytokines, including TNF-α, IL-6, CCL5, CXCL10, IFN-γ, and CCL2, were detected in the brains of JEV-infected mice (Li et al., 2015; Bian et al., 2017; Patabendige et al., 2018; Wang et al., 2018).

It was also reported that cultivation of an endothelial cell line with brain extracts obtained from JEV-infected mice downregulated the expression of tight junction proteins. In contrast, infection of isolated endothelial cells with JEV did not alter their expression or cell permeability (Li et al., 2015). These data corroborated the hypothesis that infection of endothelial cells *per se* was not responsible for BBB breakdown. Indeed, other findings suggested that the inflammatory response triggered after viral dissemination to CNS promoted BBB permeability and consequent neurological syndrome. First, JEV-infected mice presented virus RNA in the brain from 2 days post infection (dpi), whereas significant BBB disruption was only observed after 4 dpi (Li et al., 2015). Also, treatment of infected mice with neutralizing anti-IFN-γ or anti-CXCL10 antibodies preserved the BBB integrity, with no significant effect on the brain viral load (Li et al., 2015; Wang et al., 2018). Importantly, BBB breakdown was only detected in the mice that developed severe symptoms. These findings support that JEV can reach the CNS independent of BBB disruption, but subsequent events disturb the barrier, which might be a major event related to neurological disease.

Although JEV replication in the brain endothelial cells does not appear to be a major event for virus neuroinvasion, it might be relevant for the subsequent inflammation in the CNS *in vivo*. JEV infection of BMECs promoted the expression of adhesion molecules and secretion of chemokines, enhancing leukocytes adhesion (Lai et al., 2012). However, it should be noticed that leukocyte infiltration is not always deleterious to the host. In this sense, the presence of activated CD8^+^ T cells in the brains of JEV-infected mice was associated with lower mortality and preservation of the BBB integrity (Jain et al., 2017). Therefore, the profile of activated immune cells that cross the BBB and reach the brain might determine disease outcome.

## West Nile Virus

West Nile virus (WNV) was first isolated in 1937 in Uganda, and during several decades, it was associated to occasional outbreaks in Africa and in the Middle East. Since the decade of 1990, WNV spread to different countries in Europe and Americas, especially United States, and it is now considered one of the most geographically widespread arboviruses in the world. It is estimated that around 80% of the infections are subclinical and most of the symptomatic patients will develop what is called West Nile fever. However, a small proportion of the infected individuals may develop a severe or neuroinvasive disease, leading to encephalitis and meningitis (Sejvar et al., 2003).

There are some hypotheses for WNV neuroinvasion, including the hematogenous and the transneural routes (Suen et al., 2014), and increasing evidence indicate that WNV entry into the CNS is a multistep process employing different mechanism as the infection progresses. Here, we will focus in the studies evaluating CNS invasion after systemic inoculation, which supports the hematogenous pathway of virus entry.

The role of BBB disruption for WNV neuroinvasion and infection-mediated neuropathology is controversial. Several reports demonstrated that WNV infects brain endothelial cells, astrocytes, and neuron cells (Dai et al., 2008; Verma et al., 2010; Hussmann et al., 2013). Direct infection of BMECs induced degradation of tight junction proteins, in a pathway apparently dependent on their endocytosis and lysosomal degradation (Xu et al., 2012). Although endothelial barrier permeability was not directly addressed in this model, disorganization of tight junctions was proposed as a mechanism of BBB disruption and neuroinvasiveness. Other studies, however, indicated that direct infection of BMECs did not result in increased permeability. It is then proposed that viral invasion and neurovirulence are a more complex multistep process involving virus replication in different cell types, along with systemic and neuronal inflammation.

Verma et al. (2009) used an *in vitro* model of BBB, composed of primary HBMEC infected with the neurovirulent strain of WNV (NY99), and they observed that cell-free WNV crossed the endothelial barrier without affecting its integrity. Increased expression of the adhesion molecules VCAM-1 and E-selectin was observed at the peak of WNV replication, suggesting that the infection of endothelial cells might facilitate the migration of leukocytes (Verma et al., 2009). Infiltrating leukocytes could be later associated to virus entry into the brain *via* “Trojan horse” mechanism. Another study demonstrated that WNV virus-like particles (VLPs) were able to cross human umbilical vein endothelial cells (HUVEC) from the apical to the basolateral sides, suggesting that transendothelial migration of WNV may occur independent of viral replication (Hasebe et al., 2010). Comparison between VLPs from high and low virulent strains demonstrated that the first presented a higher migration efficiency in comparison to the latter. ZO-1 expression and endothelial permeability were not altered during VLP transport, and transendothelial migration was inhibited by filipin (Hasebe et al., 2010). These data support the hypothesis that WNV crosses the blood endothelial barrier, in a pathway dependent of raft-associated membrane transport, that causes no direct effect on the barrier integrity. However, this study was performed with HUVECs, and the findings need to be confirmed in brain endothelial cells.

In order to further understand the neuroinvasive differences observed between WNV strains, Hussmann et al. (2013) compared the infection rate of an avirulent WNV lineage (MAD78 strain) to a highly virulent lineage (New York strain), in neurons, astrocytes, and microvascular endothelial cells. Both strains replicated efficiently in neurons and BMECs. Also, similar levels of infectious particles were detected in the luminal and abluminal chambers of a transwell system with infected BMECs. Thus, the ability of WNV to infect the endothelial cells and to cross the BBB might not be the main responsible for the differential neuroinvasiveness of the virus strains. This diverges with the data obtained with VLP (Hasebe et al., 2010); however, different strains and distinct endothelial cell lines were used in the referred studies. Alternatively, the differences detected with VLPs from distinct strains might be overcome during a productive infection. In the study from Hussmann, the low virulent strain replicated less efficiently in astrocytes, exhibiting a delay in viral genome synthesis and reduced cell-to-cell spread in this cell type. Furthermore, in a transwell system in which infected HBMECs were seed in the luminal chamber, with astrocytes cultured in the abluminal one, there was significant lower virus expression in the astrocytes, indicating that these cells could restrain virus spread within the CNS (Hussmann et al., 2013).

On the other hand, astrocyte activation seems to participate in the rupture of BBB observed during infection. Verma et al. (2010) demonstrated that infection of HBCA astrocyte cell line with WNV upregulated the expression of MMP-1, -3, and -9 along with a downregulation of TIMP-2. Culture of microvascular endothelial cells with supernatants obtained from WNV-infected HBCA diminished the expression of claudin and ZO-1 and increased the permeability of *in vitro* BBB (Verma et al., 2010). All these events were reversed by previous treatment of the supernatants with MMP inhibitors, corroborating the idea that WNV-induced production of MMPs by astrocytes may disrupt the barrier, even when the endothelial cells are not infected.

The relevance of BBB breakdown for virus invasion of CNS, neurovirulence, and lethality of WNV *in vivo* is also controversial. Morrey et al. (2008) and Wang et al. (2004) demonstrated an increased permeability of the BBB upon WNV infection of C57BL/6 mice. In contrast, infection of BALB/c mice did not induce BBB permeability, despite leading to death (Morrey et al., 2008). Those data indicated that WNV entry in the CNS might be independent of BBB breakdown, although viral load in the brains had not been accessed in this model. The hypothesis that BBB permeability may be a consequence and not a cause of the virus entry in the CNS was supported by other *in vivo* study with C57BL/6 mice. In this model, BBB disruption upon WNV infection occurred along the course of infection and not prior to the entry of CNS. BBB breakdown resulted from decreased expression of tight and adherent junction proteins (claudin-1, occludin, ZO-1, and JAM-A; β-catenin and VE-cadherin) and correlated with the peak of WNV titers, increased production of matrix metalloproteinases (MMPs), and leukocyte infiltration in the brain (Roe et al., 2012). Importantly, intracranial inoculation of WNV also induced increased MMP expression and BBB permeability, suggesting that BBB breakdown could be induced by virus replication into the brain (Roe et al., 2012).

Mice deficient in MMP9 expression (MMP9^−/−^ KO mice) were more resistant to infection, in comparison to WT mice, despite presenting equivalent viremia and similar levels of inflammatory cytokines and IFN-α in the circulation. However, MMP9^−/−^ animals presented lower viral load in the brains, accompanied by less BBB permeability, less leukocyte infiltrates, and decreased levels of cytokines in this tissue (Wang et al., 2008a). It is important to notice that despite high viremia, no detectable permeability of the BBB was observed at early time points after infection. Clear BBB breakdown was only detected at late time points, when brain viral titer was already high (Wang et al., 2008a), indicating that virus invasion preceded BBB disruption. These findings and the *in vitro* transwell data support the hypothesis that WNV can migrate through the endothelial cells, what may allow the infection of astrocytes. Astrocytes could be then the cell source of MMP, leading to subsequent BBB breakdown and neuropathology.

Besides or along with virus replication in endothelial brain barrier, leukocyte recruitment appears to be an important event associated to BBB disruption and viral neurovirulence. As previously described, infection of endothelial cell lines with WNV induced increased expression of adhesion molecules (Dai et al., 2008; Roe et al., 2014). Lymphocytes and monocytes showed efficient adhesion and migration through a monolayer of WNV-infected HBMECs. Also, the treatment of WNV-infected endothelial cells with anti-ICAM or anti-VCAM neutralizing antibodies abolished leukocyte adhesion and migration and resulted in decreased endothelial permeability (Roe et al., 2014). Infection of C57BL/6 mice with WNV NY strain also resulted in increased expression of ICAM-1, VCAM-1, and E-selectin in the brain (Dai et al., 2008). ICAM-deficient mice showed similar systemic viral load and inflammatory cytokine levels, in comparison to WT mice. However, ICAM-1^−/−^ mice presented decreased BBB permeability, with lower leukocyte infiltration, diminished brain viral load and neuronal damage, and increased survival, in comparison to WT mice (Dai et al., 2008).

According to the referred data, it is tempted to speculate that WNV infection of BMECs might be a first essential event occurring after viremia that allows virus transmigration into the CNS. BMEC infection also induced an increased expression of adhesion molecules and production of chemokines, which could contribute to the recruitment of monocytes, lymphocytes, and polymorphonuclear cells to the barrier. The detection of augmented MMP9 expression in the brains after virus replication (Wang et al., 2008a) suggests that subsequent virus infection of other cell types may trigger a secondary BBB disruption that then allows leukocyte infiltration. The latter event would contribute for further virus invasion through a Trojan horse mechanism. This idea is supported by recent mouse experimental data showing that cerebral osteopontin stimulated the infiltration of WNV-infected neutrophils, leading to increased viral burden in the brain and mortality (Paul et al., 2017). Also, virus proteins were detected in the lymphocytes from the brain of WNV-infected mice, suggesting that infiltrating T cells might be a source of virus entering the CNS following a systemic infection (Wang et al., 2008b).

The relevance of the systemic immune response to later BBB breakdown and virus invasion was also sustained by an earlier article accessing the role of innate immune activation for WNV neural disease. The study demonstrated that TLR3-deficient mice showed lower levels of IL-6, TNF-α, and type I IFN in the blood and in the brains. TLR3-deficient mice were protected from BBB breakdown, viral invasion, leukocyte infiltration, and lethality (Wang et al., 2004), indicating that systemic inflammation was determinant for virus dissemination. After that, several studies have been investigating the role of different elements of innate immune response in the protection or enhancement of WNV neurovirulence. It was reported that signal transduction mediated by type III or type I IFN receptors was important for the maintenance of BBB integrity and for controlling WNV neuroinvasion (Lazear et al., 2015; Miner et al., 2015; Daniels et al., 2017). *In vitro* infection of different cell types demonstrated that IFN-λ addition or depletion did not significantly affect WNV replication. Still, systemic WNV infection of mice deficient in IFN-λ receptor (Ifnlr1^−/−^) resulted in higher virus concentration in the brain and spinal cord, despite similar viral load in the blood, spleen and other systemic tissues (Lazear et al., 2015). The only difference detected between WT and Ifnlr1^−/−^ mice was an increased BBB permeability in the latter. In addition, using a transwell model of BBB, it was demonstrated that IFNLR signal in BMEC increased barrier resistance. These data indicated that, although IFN-λ did not inhibit virus replication *in vitro* or *in vivo*, it increased barrier integrity and protected mice from the neurological effects caused by WNV infection. Importantly, no difference of brain viral load was detected upon intracerebroventricular inoculation of Ifnlr1^−/−^ mice with WNV, supporting the idea that the major protective role of IFN-λ was the maintenance of the barrier strength.

Type I IFN signaling pathway was also reported to be pivotal for the maintenance of BBB integrity, at least in experimental models. Daniels et al. (2014) demonstrated that addition of type I IFN to an *in vitro* culture of murine BMEC infected with WNV preserved BBB integrity and tight junction organization and reduced viral traffic through the endothelial monolayer. Also, BMECs obtained from ifnar^−/−^ mice exhibited a significant reduction in TEER and increased secretion of TNF-α and IL-1β upon WNV infection *in vitro*. It was then suggested that type I IFN might regulate the transendothelial trafficking of WNV by restraining the BBB permeability induced by those cytokines (Daniels et al., 2014). *In vivo* data corroborated these findings, since IFNAR (ifnar^−/−^) or IRF7 (irf7^−/−^) knockout mice infected with WNV presented higher BBB permeability and tight junction dysregulation compared to WT mice. Another mouse experimental model of WNV infection demonstrated that selective depletion of IFNAR in astrocytes resulted in increased BBB permeability, viral neuroinvasion, neuronal cell death, and immunopathology. Importantly, the loss of signaling *via* astrocyte IFNAR led to higher mortality rate of WNV-infected mice due to augmented virus entry, but not to virus replication into the brain (Daniels et al., 2017). These findings corroborated the *in vitro* data showing that the activation of astrocytes was determinant for BBB disruption.

Finally, the relevance of the BBB for WNV neurovirulence was accessed in mice deficient in several TAM receptors. In a study performed by Miner et al. (2015), it was demonstrated that Axl/Mertk-deficient mice exhibited increased BBB permeability and higher viral load and mortality upon subcutaneous infection of WNV, in comparison to wild-type mice (Miner et al., 2015). In contrast, no difference in viral expression in the brain was detected when the virus was inoculated directly in the CNS by intracranial injection. These findings suggest that TAM receptors exert their protective role by preserving BBB integrity and restricting virus invasion of the CNS rather than affecting neuronal replication. Interestingly, it was also showed that Mertk-depleted BMECs were less responsive to IFN-β, indicating that cooperation between TAMR and IFNAR may be a mechanism for endothelial barrier protection (Miner et al., 2015).

## Zika Virus

Zika virus (ZIKV) was first described in Uganda, in 1947, and the first human cases were reported in 1963 (Dick et al., 1952; Simpson, 1964). Very few cases of human infection had been described in Africa and Asia until 2007, when an outbreak occurred at Yap Islands. Another outbreak at French Polynesia was reported in 2013, and from 2015 to 2016, a huge epidemic started in Brazil and spread throughout Americas (Duffy et al., 2009; Musso, 2015; Bogoch et al., 2016; Faria et al., 2016). Since then, ZIKV infection was associated with microcephaly and other neurological manifestations in fetuses and newborns upon vertical transmission, and this is now collectively called Zika congenital syndrome (Melo et al., 2016; Oliveira Melo et al., 2016). Although less frequent, neurological disorders have also been reported in adults, including encephalitis and Guillain-Barre syndrome (Brasil et al., 2016; Carteaux et al., 2016). ZIKV was detected in the post-mortem brains of microcephalic fetuses or stillborns and in the CSF of encephalitic adult (Carteaux et al., 2016; Mlakar et al., 2016), indicating that the virus invades the CNS. Indeed, numerous studies indicated that ZIKV is neurotropic. Viral particles and RNA were detected in the brain of mice inoculated by different pathways (Cugola et al., 2016; Li et al., 2016); in brain organoids infected *in vitro* (Garcez et al., 2016); and in the CSF of infected rhesus monkeys (Dudley et al., 2016). However, the mechanisms associated to brain invasion are still unclear.

Previous evidence indicated that endothelial cells from the BBB are permissive to replication of different ZIKV strains and that the virus may cross the endothelial barrier, without inducing a significant increase in its permeability (Shao et al., 2016; Al-Obaidi et al., 2017; Bramley et al., 2017; Mladinich et al., 2017; Papa et al., 2017; Alimonti et al., 2018). Mladinich et al. (2017) showed that ZIKV (Porto Rico strain PRVABC59) infected and replicated efficiently in primary human brain microvascular endothelial cells (HBMECs). ZIKV was basolaterally released in a transwell system, without inducing a remarkable cytopathic effect (Mladinich et al., 2017). Similarly, Papa et al. (2017) demonstrated that infection of HBMECs with African and Brazilian ZIKV strains (ZIKV_MR766_ and ZIKV_PE243_) resulted in efficient virus replication and basolateral release of infectious particles, which were able to replicate in other cell types seeded separately in a transwell system. It is important to notice that infection with ZIKV_PE243_ strain did not induce a remarkable CPE and no cell death was detected in the cultures, whereas infection with ZIKV_MR766_ was associated to some level of cytopathic effect. The viruses crossed the endothelial barrier with no apparent increase in permeability, which was further confirmed by the maintenance of tight junction protein expression and localization (Papa et al., 2017). The study suggested that ZIKV might cross the endothelial barrier by endocytosis and exocytosis-dependent replication pathway or by transcytosis. Infection with both strains also induced HBMEC activation and secretion of IL6 and CCL5, what may contribute to the recruitment of leukocytes in an *in vivo* fashion (Papa et al., 2017). Other studies that used brain endothelial cells (i-BMECs) derived from pluripotent stem cell (iPSC) also confirmed that ZIKV (Canadian isolate) infected and traversed the iBMECs, without compromising the BBB (Alimonti et al., 2018). On the other hand, a 3D cell culture system using an HBMEC cell line showed a certain resistance to infection in comparison to conventional 2D culture. However, pretreatment of the cultures with inflammatory cytokines, such as TNF-α, increased virus replication and disorganization of the junctional network, indicating that an inflammatory response triggered *in vivo* might contribute to BBB disruption and ZIKV invasion of the brain. This study also established a transwell culture, in which HBMECs were seeded in the inserts and astrocytes in the abluminal chamber. Using this system, it was demonstrated that HBMECs allowed the migration of infected monocytes, which then enhanced the infection of the astrocytes (Bramley et al., 2017).

*In vivo* experimental models supported the hypothesis that ZIKV may cross the BBB without severe disruption. In the study from Papa and colleagues using IFNAR-deficient mouse model, the presence of virus RNA was noticed very early after systemic infection, when no BBB disruption had been detected (Papa et al., 2017). Immunostaining revealed the presence of ZIKV envelope protein in blood vessels and in the cells of plexus choroid of subventricular zone, suggesting that brain endothelial cells were indeed infected by the virus. Interestingly, barrier breakdown was detected at later time points upon infection. Given that leukocytes recruitment to the brains seems to be determinant for neuronal lesion and lethality induced by ZIKV (Jurado et al., 2018), one can speculate that later BBB disruption was possibly triggered by the inflammatory response that followed virus replication. Another study comparing different ZIKV strains revealed that, depending on the mouse age, infection with ZIKV_MR766_, but not with ZIKV_PE243,_ resulted in mouse death (Lucas et al., 2018). Evidence of BBB breakdown, including endogenous IgG leakage in the brains and microhemorrhagic lesions, were only detected after infection with ZIKV_MR766_. However, it is still not clear whether BBB breakdown was essential for the neurological disease and death or if it was a marker and a consequence of a severe disease.

It should be considered that the African strain ZIKV_MR766_ was extensively passaged in mouse brains when it was isolated, what could explain its increased replication in the mouse model. Indeed, there are extensive differences between the sequences of ZIKV_MR766_ and the Asian isolates, like ZIKV_PE243_, which were not mouse adapted. Therefore, comparison between different ZIKV isolates might clarify the molecular mechanisms associated to disease or protection, including the contribution of the sequence differences for virulence, tissue tropism, pathology, and immune evasion. Regarding this, the protective response detected after infection with ZIKV_PE243_ strain was associated to specific CD4^+^ T cells and neutralizing antibody (Lucas et al., 2018), suggesting that a systemic response may affect the amount of virus reaching the BBB and the lesion extension, ultimately affecting the disease outcome.

Jurado and colleagues investigated the role of IFNAR deficiency in hematopoietic and non-hematopoietic cells in a mouse model of ZIKV infection. It was demonstrated that IFN response in non-hematopoietic cells was essential for protection against viral dissemination to the brain, BBB disruption, and ZIKV-induced neuropathology. Virus-induced paralysis was dependent of CD8^+^ T cells infiltration in the brains, indicating that leukocyte infiltration due to BBB disruption might be a major issue for neuropathogenesis. They also observed that astrocytes were the main cell types infected in the brains of susceptible mice (Jurado et al., 2018). These findings indicated that astrocyte infection after virus crossing through BMECs might be an important contributor of subsequent BBB disruption and lymphocyte infiltration.

ZIKV infection during the embryonic period also impacted vascular function, particularly the BBB (Shao et al., 2016). Inoculation of C57BL/6J or 129S1/SvImJ mice at the embryonic day 14.5 affected mouse neurovascular development, resulting in postnatal microcephaly and brain damage. Analysis of the brains at postnatal period indicated a significant increase in vessel density and diameter in the cerebral cortex, with evidence of leaky blood-brain barrier (BBB). Furthermore, the brains exhibited extensive microglial activation, astrogliosis, and high levels of IL-1β and TNF-α (Shao et al., 2016). Thus, an excessive immune response can also harm the neurovascular development, leading to increased BBB permeability and brain damage.

## Dengue Virus and Yellow Fever Virus

Dengue virus (DENV) and Yellow fever virus (YFV) viruses are usually associated to systemic and hemorrhagic clinical syndromes, and neurological manifestations are considered sporadic. Although very rare, immunization with YFV may induce encephalitis, and even though it is usually controlled by the host, it represents a clinical concern (Jennings et al., 1994; Monath and Barrett, 2003; Beck and Barrett, 2015; Halstead and Cohen, 2015; Screaton et al., 2015; Li et al., 2017).

YFV neurovirulence has been mostly investigated using laboratory neuroadapted strains and performing intracerebral inoculation in mice; therefore, the exact mechanism through which the virus accesses the brain and whether the viruses are capable to disrupt the blood-brain barrier remains unclear. Adult mice are resistant to systemic inoculation of YFV, and brain damage was only detected when high virus doses were inoculated. For this reason, neurological studies were mostly performed using intracerebral inoculation (Barrett and Gould, 1986; Liu and Chambers, 2001). Based on these models, it was suggested that YFV accesses the brain tough hematogenous route (Mims, 1957; Luethy et al., 2016). More recent studies have been using mice deficient in IFNAR and/or IFNLR, which are highly susceptible to infection (Luethy et al., 2016; Douam et al., 2017). Douam et al. (2017) demonstrated that IFNAR-deficient mouse presented increased viremia and high viral load in systemic organs, including liver, spleen, and kidney. The viral load in the brain was also higher than the one observed in WT mice, but it did not increase overtime. IFNAR^−/−^ mice also lost weight and displayed clinical manifestations; but both parameters were recovered at later time points after inoculation. In contrast, mice deficient in both IFNAR and IFNLR (ifnar^−/−^ ifnlr^−/−^) succumb to infection. These mice showed enhanced systemic infection and progressive increase of viral load in the brains, associated with evidence of BBB leakage. In both mice models, BBB permeability was only accessed at 5 dpi, whereas virus was already detected at 3 dpi; therefore, it is difficult to determine whether BBB disruption was necessary for virus invasion. Importantly, significant differences in the profile of T cells activation, especially serum IFN-γ levels appeared to influence the disease outcome (Douam et al., 2017).

Regarding DENV infection, increasing number of reports has been demonstrating the potential risk of neurological manifestations, with an incidence rating varying from 0.5 to 20% (Li et al., 2017; Vieira et al., 2018). Encephalitis was reported in children and adults, and virus RNA and anti-DENV IgM were detected in the CSF (Lum et al., 1996; Domingues et al., 2008; Vieira et al., 2018). Analysis of clinical samples obtained from dengue fatal cases also demonstrated the presence of virus RNA or proteins in the CSF and in brain sections, suggesting that DENV may indeed achieve the CNS (Miagostovich et al., 1997; Ramos et al., 1998; Araújo et al., 2011).

Similar to what was observed with other neurotropic viruses, DENV (serotype 2) was able to infect human BMECs, leading to the release of high titers of infectious particles and cell death *in vitro* (da Conceição et al., 2013). hBMEC infection was associated to cellular activation with increased expression of ICAM and enhanced secretion of inflammatory cytokines and chemokines, what may contribute to the recruitment of leukocytes *in vivo* (da Conceição et al., 2013). In another study, Velandia-Romero et al. (2016) evaluated the infection and permeability of primary mouse brain endothelial cells (MBECs), cultured either isolated or with primary astrocytes. The cells were infected with a DENV-4 isolate or with its variant, the neuroadapted strain D4MB-6. It was observed that both DENV strains infected MBECs, but not the astrocytes. MBECs infection altered the structure and function of BBB, affecting the endothelium permeability and the localization of the tight junction proteins ZO-1 and Claudin-1. TJ disorganization then allowed paracellular passing of free virus particles in a transwell system (Velandia-Romero et al., 2016). Microglia was also demonstrated to be susceptible to infection to all DENV serotypes. *In vitro* infection of murine microglia cell line (BV2) with DENV 1–4 resulted in increased expression of proinflammatory cytokines, including TNF-α, IFN-γ, IL-1β and IL-10, MCP-1, and IL-6, and of MMP-2 and MMP-9 (Bhatt et al., 2015). Although it had not been addressed, activation of microglia might contribute to BBB lesion. According to these data, infection of endothelial cells by DENV seems to be more relevant for BBB disruption, when compared to infection by other flaviviruses. Still, virus interaction with other cell types associated to BBB could also affect the barrier integrity.

A major caveat for the study of dengue pathogenesis *in vivo* is the limited susceptibility of small animal models to most DENV isolates. To overcome this issue, seminal papers were developed with mouse-adapted DENV2 strains (D2S10 and D2S20) derived from the parental PL046 isolate (Shresta et al., 2006; Orozco et al., 2012; Prestwood et al., 2012). Immunodeficient mice inoculated with D2S10 strain by systemic routes (i.v. or s.c.) presented virus in neuronal and non-neuronal tissues, showed high levels of inflammatory cytokines, and increased vascular permeability, resembling the main features of severe dengue. Importantly, mouse infection with this strain was lethal with no apparent signs of paralysis (Shresta et al., 2006; Orozco et al., 2012; Prestwood et al., 2012). On the other hand, the mice inoculated with PL046 presented virus only in the brain and spinal cord and exhibited paralysis (Shresta et al., 2006). However, the molecular mechanisms of virus dissemination to the CNS were not addressed in these models.

DENV infection in immunocompetent mice was mostly performed by intracranial inoculation. Those studies were important to evaluate the virus neurotropism but did not access the role of BBB integrity for DENV-induced CNS manifestations. Chaturvedi et al. (1991) were some of the first to report that DENV can destabilize the blood-brain barrier *in vivo*, using an experimental model of mice infected with a DENV2 virus strain. A remarkable increase in BBB permeability and high virus titers in the brains were evidenced when the mice were intracerebrally inoculated. In contrast, systemic (i.p.) infection induced slight alteration in the BBB permeability at late time points after infection, but no virus particles were detected (Chaturvedi et al., 1991). Another study based on intracranial inoculation of different virus isolates indicated that DENV serotype and genotype features may also influence the neurological disease outcome (Souza et al., 2013). However, BBB integrity was not evaluated in this model.

A major increment in the study of DENV neuropathogenesis came from the findings that mice deficient in IFN-α/βR and IFN-γR are highly susceptible and develop high viremia, paralysis, and death (Sarathy et al., 2015). Comparison between A129 (IFN-α/βR −/−) and AG129 (IFNα/β/γR −/−) mice intravenously inoculated with DENV2 demonstrated that IFN-γ signaling was essential to protect the CNS from viral invasion, through a CD8^+^T cell-mediated response (Prestwood et al., 2012). Mice deficient in IFN-α/βR / IFN-γR / FcγRIIB were also susceptible to systemic infection, and virus was detected in the brains at late time points after infection (Dhole et al., 2016). Although DENV has been extensively detected in the brains and spinal cord upon systemic infection of AG129 mice, the pathway through which flaviviruses reach the CNS was not addressed (Shresta et al., 2004; Prestwood et al., 2012).

Velandia-Romero et al. (2012) developed another model of DENV infection *in vivo*, using suckling BALB/c mice (2–21 postnatal days) infected with the neuroadapted strain of DENV-4 (D4MB-6). Intraperitoneal inoculation of mice at 14 or 21 days after birth did not result in any clinical signs or mortality. In contrast, mice inoculated at 2 and 7 postnatal days developed fatal encephalitis accompanied by paralysis and postural instability. BBB leakage and increased viral replication in the brains were detected and correlated with the clinical outcome (Velandia-Romero et al., 2012). However, BBB integrity was only evaluated at 6 days post infection, whereas virus replication was already detected in the brains at 3 dpi. Therefore, it is hard to determine whether the barrier disruption was essential or not for virus invasion of the CNS. Importantly, DENV antigen was detected in endothelial cells and in the microglia of the mice that developed neurological manifestations. Activated phenotype of the infected cells, astrocytosis, and leukocyte infiltration, close to hemorrhagic focuses, were also evidenced (Velandia-Romero et al., 2012). These findings suggested that virus-BBB interaction might be relevant to DENV neuropathogenesis.

DENV dissemination was also accessed in a primate model of DENV infection with antibody-dependent enhancement (ADE) (Vasconcelos et al., 2016). *Callithrix penicillate* primates were subcutaneously infected with DENV3, followed by treatment with anti-DENV2 antibodies to mimic ADE. After several inoculations, virus antigen and microglia activation were detected in the brains, along with evidence of inflammation. Although the infection by DENV in the absence of ADE had not been explored, these findings supported the hypothesis that DENV is able to reach the CNS. However, infection of brain endothelial cells or BBB integrity was not investigated in this model.

All the referred data indicate that DENV may potentially infect brain endothelial cells, activate those and other cells associated to the BBB, and cause neurological manifestations. However, except for the few studies with human fatal cases, most findings were achieved with limited experimental models. The use of small animal models is an alternative to bypass the technical complications of human studies. However, it has been largely demonstrated that different flaviviruses evade interferon response in human but not in mouse cells (Ashour et al., 2010; Grant et al., 2016). Therefore, the dissemination of these viruses to the CNS after systemic infection has been mostly observed in immunodeficient mice. Although the relative resistance of adult wild-type mice to neurological disease caused by flaviviruses is a major caveat, some researchers claim that IFNAR-deficient mice might be a relevant model, since the IFN response in human cells is normally counteracted by these viruses. Still, there are several gaps in the comprehension of the pathway through which DENV reaches the brain that worth to be investigated.

## Tick-Borne Encephalitis Virus

Tick-borne encephalitis virus (TBEV) is part of another complex of flaviviruses, which are not transmitted by mosquitoes, and it is present in Europe and Asia, with an estimation of 10,000 to 15,000 cases per year (Kellman et al., 2018). Most infections are asymptomatic or are associated to a mild flu-like syndrome. However, in some individuals, the initial symptoms are followed by a second phase, characterized by neurological manifestations ranging from mild meningitis to severe encephalomyelitis. Up to 40% of these cases result in long-term neurological sequelae following encephalitis (Holzmann, 2003).

Like the other flaviviruses described here, the pathogenesis of tick-borne encephalitis in humans is not completely determined, but *in vitro* and *in vivo* infection models have been unveiling some aspect of the infection. Once the TBEV reaches the CNS, neurons are believed to be the primary target of virus replication. *In vitro* assays demonstrated that the virus is cytopathic and productively replicate and disseminate in primary human neurons and in human neural cell lines (Růzek et al., 2009; Bílý et al., 2015). Astrocytes were also reported to be susceptible to TBEV replication, but resistant to virus-induced cytopathic effect. Rat primary astrocytes were productively and persistently infected by TBEV, but no cytotoxicity was observed until 14 dpi (Potokar et al., 2014). Similarly, Palus et al. (2014) demonstrated that infection of primary human astrocytes (HBCAs) by TBEV resulted in sustained intracellular viral load and release of infectious particles, with no CPE or cell death until 15 dpi. Despite the lower frequency of infected cells in the culture, astrocyte activation was clearly evidenced by increased expression of glial fibrillary acidic protein (GFAP), higher mRNA expression of inflammatory cytokines and chemokines, and enhanced production of MMP-9 (Palus et al., 2014). These findings suggested that astrocytes infection with TBEV could affect BBB integrity by the release of proinflammatory cytokines and MMP. However, like other neurotropic viruses, TBEV induced the expression of type I IFN in astrocytes, which restricts the infection efficiency. Abrogation of IFN response by using astrocytes from IFNAR-deficient mice or by adding neutralizing antibodies to the cultures rendered mouse astrocytes more susceptible to TBEV infection, with increased virus replication and dissemination, and diminished cell viability (Lindqvist et al., 2016). In addition, infection of cells in the presence of supernatants obtained from infected astrocytes induced ISG expression and limited TBEV infection, supporting the hypothesis that IFN released by astrocytes upon TBEV infection might limit viral dissemination (Lindqvist et al., 2016).

Still, the pathways by which TBEV reaches astrocytes and neurons were not determined. It was only recently demonstrated that TBEV replicate in human BMECs (Palus et al., 2017). *In vitro* assays comparing lower and higher neuroinvasive lab strains of TBEV demonstrated that both strains productively replicated in HBMECs but did not induce remarkable CPE at the time points analyzed (Palus et al., 2017). Importantly, TBEV infection did not affect the expression or localization of tight junction proteins nor the expression of adhesion molecules. Also, the viruses were able to transmigrate through the endothelial barrier *in vitro*, without enhancing its permeability (Palus et al., 2017).

*In vivo* experimental mouse models demonstrated that subcutaneous infection with TBEV promoted neurological signs and death (Růžek et al., 2011). Kinetic analysis of virus dissemination and BBB integrity indicated that infectious particles were detected in the brain only after 7 dpi, simultaneously with the decrease of viremia. BBB disruption was not observed during the viremic phase of the infection, and it was only detected after 10 dpi. At this time point (10 dpi), the clinical symptoms were most evident, and the mice started to die. Taken together, these data indicated that infection of endothelial cells by TBEV does not affect the integrity of BBB. Also, although virus-endothelial cell interaction might be relevant for virus transmigration and invasion of the CNS, these events seem to not require a significant disruption of the BBB. The impact on the BBB structure is possibly rather a consequence of the brain infection, but it is still determinant for disease severity (Růžek et al., 2011).

## Concluding Remarks

Several flaviviruses are potentially neurotropic and neurovirulent, but the knowledge regarding the mechanisms of neuropathogenesis is very incipient for most of them. Also, the pathway through which flaviviruses reach the CNS is still unclear. Given the systemic nature of the initial infections caused by JEV, WNV, ZIKV, DENV, YFV, and TBEV, it is believed that they reach the brain mainly through a hematogenous route. Indeed, hemorrhagic focuses or other evidence of BBB leakage are often observed upon infection, indicating that viruses might cross the endothelial brain barrier to reach the CNS and cause neurological syndromes. *In vitro* experimental models have demonstrated that different viruses productively infect brain endothelial cells and induce cell death, what could be a major contributor to BBB damage and virus crossing. However, the role of BBB disruption for viral invasion is controversial, and increasing evidence suggest that flaviviruses can traverse the endothelial barrier by transcellular pathways. In either case, viruses could then access other cells associated to the BBB, such as pericytes, astrocytes, and microglia. Infection of these cells might induce cellular activation and release of proteases and other mediators that affect TJ organization and BBB integrity. Barrier breakdown could be, then, a secondary effect due to the inflammatory response into the brain. Still, BBB damage seems to be essential for neuropathology, by further increasing the local viral load, leukocyte infiltration, and inflammation. Different pathways proposed for flaviviruses entering the brain through the BBB are depicted in Figure 1. The scheme suggests that flaviviruses may reach the CNS by cell-free virus transcytosis through the endothelial cells or by transmigration of infected leukocytes into the CNS. Both mechanisms would allow the release of infectious virus into the brain that could then infect BBB-associated cells and neurons. Either pathway will be followed by an inflammatory response. Inflammation, virus replication in endothelial cell, and/or cells associated to the barrier would then trigger BBB leakage.

It is important to consider that most of the studies regarding the role of endothelial cell infection and BBB disruption for neuroinvasion and disease were performed *in vitro* or in mouse experimental models. *In vitro* assays were fundamentally developed using human transformed cell lines or primary cells from rodents, which are not natural hosts of these infections. Furthermore, wild-type adult mice usually control systemic infection by flaviviruses and do not develop neurological manifestation. For this reason, *in vivo* assays aimed to explore neurological syndromes were formally performed by intracranial inoculation. Systemic inoculation and investigation of virus invasion to the CNS in experimental models have been restricted to immunodeficient or suckling mice. Those are susceptible to virus dissemination, invasion of the CNS, and neuronal damage. It is important to point that even in these models, the phenomena are usually evaluated at fixed time point after infection, which does not give a clear picture of the sequence of events preceding CNS damage. Also, a detailed analysis of the specific cells infected by the viruses was poorly performed in *in vivo* models.

The investigation regarding flavivirus neuroinvasion upon natural human infection is also very incipient. It probably reflects the inefficient or delayed diagnosis of the patients, which restricts the number of available samples with confirmed infection by flaviviruses. Clear investigation of the cells infected in the blood-brain barrier and CNS parenchyma requires good quality pathological analysis or cell isolation, followed by phenotypic or molecular evaluation of infection and activation. The inherent difficulty for tissue obtention for these analyses, associated to the low number of available samples, has been restricting the research to molecular diagnosis and confirmation of virus invasion of the CNS. Hence, whether brain endothelial cells are targeted by flavivirus infection and whether this event, associated or not with BBB breakdown, is relevant for virus invasion and neuropathogenesis upon natural human infection are still opening questions in the field.

Finally, the experimental models have been unveiling several specific issues regarding flavivirus neuroinvasion, significantly improving the knowledge about these neglected diseases. However, systematic experiments designed to evaluate whether brain endothelial cells are indeed infected *in vivo* and to investigate the kinetics and relevance of the BBB breakdown are still need to be performed. Also, effort should be taken to improve flavivirus diagnosis and to strengthen the interaction between physicians and researchers, aiming to better understand the events followed flavivirus infection that leads to neuroinvasion and neurologic diseases in humans. Full comprehension of these mechanisms would identify specific molecular targets and stimulate the development of new medications against flavivirus neurological syndromes, ultimately benefitting the patients.

## Author Contributions

YM, LM, SC, and LA wrote the review. LA revised the figure and final text.

### Conflict of Interest Statement

The authors declare that the research was conducted in the absence of any commercial or financial relationships that could be construed as a potential conflict of interest.
